# Citation Classics and Trends in the Field of Opioids: A Bibliometric Analysis

**DOI:** 10.7759/cureus.5055

**Published:** 2019-07-01

**Authors:** Hira F Akbar, Khadijah Siddiq, Salman Nusrat

**Affiliations:** 1 Internal Medicine, Dow Medical College, Dow University of Health Sciences, Karachi, PAK; 2 Internal Medicine, Civil Hospital Karachi, Dow University of Health Sciences, Karachi, PAK; 3 Gasteroenterology, University of Oklahoma Health Sciences Center, Oklahoma City, USA

**Keywords:** citation classics, opioids, bibliometric analysis, scopus, citescore

## Abstract

Introduction

Bibliometric analysis is one of the emerging and latest statistical study type used to examine and keep a systemic record of the research done on a particular topic of a certain field. A number of such bibliometric studies are conducted on various topics of the medical science but none existed on the vast topic of pharmacology - opioids. Hence, we present a bibliometric analysis of the ‘Citation Classics’ of opioids.

Method

The primary database chosen to extract the citation classics of opioids was Scopus. Top 100 citation classics were arranged according to the citation count and then analyzed.

Results

The top 100 citation classics were published between 1957 and 2013, among which seventy-two were published from 1977 to 1997. Among all nineteen countries that contributed to these citation classics, United States of America alone produced sixty-three classics. The top three journals of the list were multidisciplinary and contained 36 citation classics. Endogenous opioids were the most studied (n=35) class of opioids among the citation classes and the most studied subject was of the neurosciences.

Conclusion

The subject areas of neurology and analgesic aspects of opioids are well established and endogenous and synthetic opioids were the most studied classes of opioids. However, the egregious issues of addiction and misuse of opioids were underrepresented in the citation classics. The pulmonary and gastrointestinal aspects of opioids are also marginalized among the citation classics.

## Introduction

Each year, extensive contributions are made to the medical science literature [1]. Bibliometric research provides quantitative analysis and extensive insight regarding the research conducted, the era in which the work is performed, and eminent authors, countries, and institutions; this approach also identifies aspects that remain to be examined [2], hence to monitor studies regarding a specific topic, practitioners refer to bibliometric researches. Although several bibliometric studies have been conducted in breast cancer [3], orthopedic surgery [4], epilepsy [5], thrombolytic therapy [6], and valvular heart diseases [7], our literature search revealed no such bibliometric analysis for opioids.

Opioids are the prototypical derivatives of opium, which is considered one of the world’s oldest drugs [8]. Opioids are extensively used as analgesics, antitussive agents, antidiarrheal drugs, and anesthetic agents for management of acute pulmonary edema and various other treatments [9]. Some opioids, such as heroin, are illegal [10]. Moreover, the abuse or overuse of opioids can result in adverse effects and opioid dependence [11].

To fill this gap, we conducted a bibliometric analysis on opioids. The paper presents the opioid citation classics, that is, articles that were cited more than 400 times [12], as well as the trends in recently published articles.

## Materials and methods

A citation search was conducted to identify the 100 most cited articles and citation classics in the available literature (see Appendix). Like other researchers [6,7], we chose Elsevier’s Scopus online database for our search, as Scopus provides 20% more coverage than Web of Science with more accurate citation counts than Google Scholar [13]. Full articles were accessed from PubMed, EMBASE (Excerpta Medica dataBASE), and Science Direct.

‘Opioids’, ‘opiates’, and ‘opium’ were our primary search keywords. To avoid bias and prevent missing relevant articles, we expanded our list to the names of individual opioids mentioned in Basic and Clinical Pharmacology [14]. The extended list included ‘buprenorphine’, ‘butorphanol’, ‘codeine’, ‘diphenoxylate’, ‘fentanyl’, ‘heroin’, ‘levorphanol’, ‘loperamide’ ‘methadone’, ‘meperidine’, ‘morphine’, ‘naloxone’, ‘nalbuphine’, ‘nalmefene’, ‘naltrexone’, ‘oxycodone’, ‘propoxyphene’, ‘pentazocine’, ‘tramadol’, and ‘tapentadol’. All electronic database searches were performed on October 9th, 2017. Keywords were searched in ‘article titles’, ‘abstracts’, and ‘keywords’. Relevant articles were retrieved and sorted by the option of ‘Cited by’, which yielded a list of articles arranged in descending order of their number of citations. No filters of language, time, human studies, subject area, territory, or affiliations were used. Abstracts and full texts of the articles were read from the sorted list and irrelevant articles were excluded from the analysis.

All article types, other than those requiring manual searching, telephone access, guidelines, and non-PubMed indexed articles, were included. The dataset was further evaluated by title, first and senior author, institution, department of the first author, topic, source, year of publication, and country of origin. In contrast with other researchers [6,7], we used CiteScore [15], Source Normalized Impact per Paper (SNIP), and SCImago Journal Rank (SJR) to rank the journals. Some articles were cited more frequently than others, due to differences in the time since publication. We adjusted for this error by determining citation index for each article.

Citation analysis of the extracted articles was conducted both by using Scopus and by manually screening the articles. Articles were further classified into two broad categories: 1) major subject area and 2) class of opioids. Tables and charts were created using Microsoft Excel 2016 (Microsoft Corporation, Washington, US). IBM Statistical Package for the Social Sciences (IBM SPSS Statistics for Windows, Version 20.0. Armonk, New York, US) was used to apply the Pearson product moment correlation co-efficient to evaluate the relationship between citation times, CiteScore, and citation density. Chi-square test was applied to observe significant difference between the categorical values and Non-parametric tests, such as the Mann-Whitney U test and Kruskal-Wallis test, were applied to evaluate any significant difference between categorical and numerical data. P-value < 0.05 was considered significant in all cases.

## Results

Trends of citation classics

**Figure 1:**
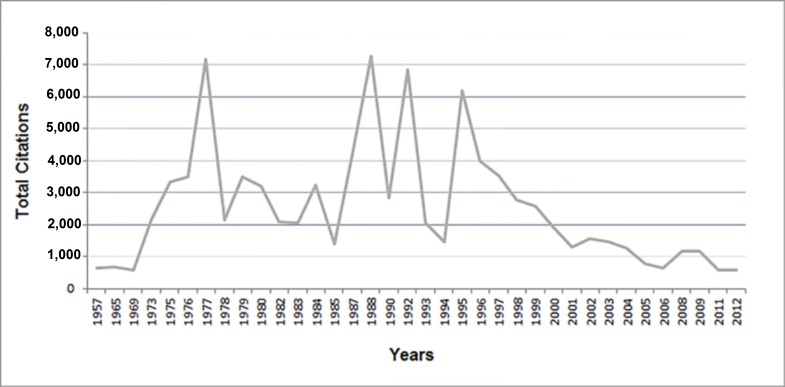
Total citations per year.

Top citation classics were published from 1957 to 2013. Most were original articles (n=80), while the remainder were review articles (n=17), conference papers (n=1), and letters to the editor (n=2). The citations of those articles summed to 92,413, ranging from 545 to 3,149, with a median of 762.5 and a mean of 924.13. Approximately 4% were self-cited and 3.6% were book citations, reducing the original citations to 85,482. Citation density (citations per year) ranged from 10.53 to 147.63, with a median of 33.06 and a mean of 38.97. A moderately positive correlation was found between citation count and citation density (r=0.511), while no significant correlation was found between citation count and year of publication. There was no significant difference between the citations of original articles and other article types (p= 0.311). Figure 1 shows the overall trend of total citations by year, with the peak citations in 1988.

Origins, institutions and authorships

**Figure 2:**
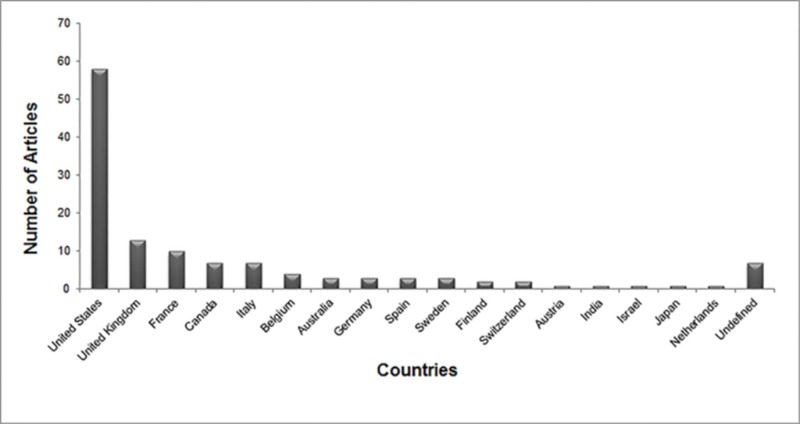
Articles originating from each country.

The citation classics were produced by nineteen different countries. Figure 2 shows major contributors to the top 100 citation classics, with the leading contribution from the United States (n= 58).

In addition, 160 different institutions contributed to the top cited articles list, with almost 40 institutions having two or more articles in the list. The University of Michigan (n=15) and VA Medical Center (n=7) had the most citation classics of all institutions on the list. The third most-cited institution, University of Cagliari (n=5), is in Italy. 

Five hundred authors contributed to the citation classics, with a median of four authors and mean of five authors. The number of authors per article ranged from 1 to 21. Details of authors with more than two citation classics are shown in Table 1.

**Table 1:** Authors with more than two articles in the top 100.

Author	Number of Articles	Author Position				Author Affiliations	H index	Primary topic of interest	Fields of Interest	Years	Highest Citation
		First	Last	Others	Corresponding						
Akil, H	6	1	1	4	0	Molecular & Behavioral Neuroscience Institute, University Michigan Ann Arbor, Ann Arbor, United States	101	Receptors/ Anatomy	Neuroscience, Genetics and Biochemistry	1984-1995	1,006
Watson, SJ	5	0	4	1	0	Molecular & Behavioral Neuroscience Institute, University Michigan Ann Arbor, Ann Arbor, United States	108	Receptors/ Anatomy	Neuroscience, Genetics and Biochemistry	1984-1995	954
Di Chiara, G	4	2	2	0	2	Consiglio Nazionale delle Ricerche, Institute of Neuroscience, Roma, Italy	78	Addictions and Drug abuse	Neuroscience, Pharmacology and Biochemistry	1988-1997	3,149
Mansour, A	4	4	0	0	4	Pharmaco Genesis, United States	40	Receptors/ Anatomy	Neuroscience, Genetics and Biochemistry	1987-1995	954
Hughes, J	3	2	0	1	2	The Neuroscience Research Centre, Harlow, United Kingdom	40	Drug interaction and pharmacology	Neuroscience, Medicine and Pharmacology	1975-1977	2,677
Imperato, A	3	0	2	1	0	Sanofi S.A., Neurodegenerative Disease Program, Gentilly, France	44	Addictions and Drug abuse Drug interaction and pharmacology	Neuroscience, Genetics and Biochemistry	1988-1999	3,149
Kalso, E	3	1	0	2	0	Helsinki University Central Hospital, Intensive Care and Pain Medicine, Helsinki, Finland	64	Pain Management	Neuroscience, Medicine and Pharmacology	2001-2004	969
Kosterlitz, HW	3	0	2	1	0	University of Aberdeen, Aberdeen, United Kingdom	101	Receptors/ Anatomy, Pharmacology	Pharmacology, Biochemistry and Genetics	1975-1983	2,677
Ling, N	3	0	1	2	0	Neurocrine Bioscience, Department of Peptide Chemistry, San Diego, United States	96	Pharmacology and endorphins	Neuroscience, Medicine and Biochemistry	1976-1977	1,101
Roques, BP	3	1	0	2	1	Pharmaleads, Paris, France	82	Pharmacology and opiums	Neuroscience, Pharmacology and Biochemistry	1980-1999	1,139
Vassart, G	3	0	0	3	0	Université libre de Bruxelles (ULB), Institut de Recherche Interdisciplinaire en Biologie Humaine et Moléculaire (IRIBHM), Brussels, Belgium	90	Receptors/ Anatomy	Biochemsitry, Medicine and Pharmacology	1994-1999	1,636
Bloom, F	3	1	2	0	1	Scripps Research Institute, San Diego, United States	117	Pharmacology and endorphins	Neuroscience, Genetics and Biochemistry	1976-1988	1,606

Journals

The citation classics were published in 40 journals. The CiteScore of these journals ranged from 2.45 to 29.6, with a median of 8.67 and a mode of 7.87. More than half of the articles (n= 62) were published in nine journals (Table 2). Weak positive correlations were found between CiteScores of the journals and the number of articles published (r=0.255), total citations of the journals (r=0.253), citation count of the articles (r=0.080), citation density (r=0.085), and years of publication (r=0.086). There was no significant difference between CiteScore by subject area (p=0.432), classification of opioids (p=0.076), or type of article (p=0.267).

**Table 2:** Journals with more than two articles in the top 100. ^ⱡ^SNIP: Source Normalized Impact per Paper. ^₴^SJR: SCImago Journal Rank

SOURCE TITLE	Number of Articles	CiteScore	Highest CiteScore Percentile	SNIP^ⱡ^	SJR^₴^	% review	%cited	Total citations	CiteScore Rank	Subject Area
Science	14	14.39	99th	13.53	7.68	10.83	34.83	13,914	1/77	Multidisciplinary
Nature	12	13.33	98th	8.04	18.13	2.02	42.97	12,720	2/77	Multidisciplinary
Proceedings Of The National Academy Of Sciences Of The United States Of America	10	8.56	96th	2.63	6.32	1.16	15.76	9,883	3/77	Multidisciplinary
Anesthesiology	6	3.01	91st	2.27	2.12	7.88	51.45	4,373	2/23	Anesthesiology and Pain medicine
Pain	5	4.58	97th	1.94	2.84	8.09	27.75	5,437	3/115	Anesthesiology and Pain medicine
Journal Of Pharmacology And Experimental Therapeutics	4	3.9	85th	1.16	1.76	3.54	19.52	4,689	43/299	Pharmacology, Toxicology and Pharmaceutics
JAMA Journal of American Medical Association	4	6.67	98th	9.16	6.86		48%	3,557	23/2156	Medicine
New England Journal Of Medicine	4	12.82	99th	14.68	15.76	10.12	54.74	3,850	7/2156	Medicine
Brain Research	3	2.75	78th	0.82	1.26	19.53	29.61	1,942	69/322	Neurology

Subject areas and topical distribution

**Figure 3:**
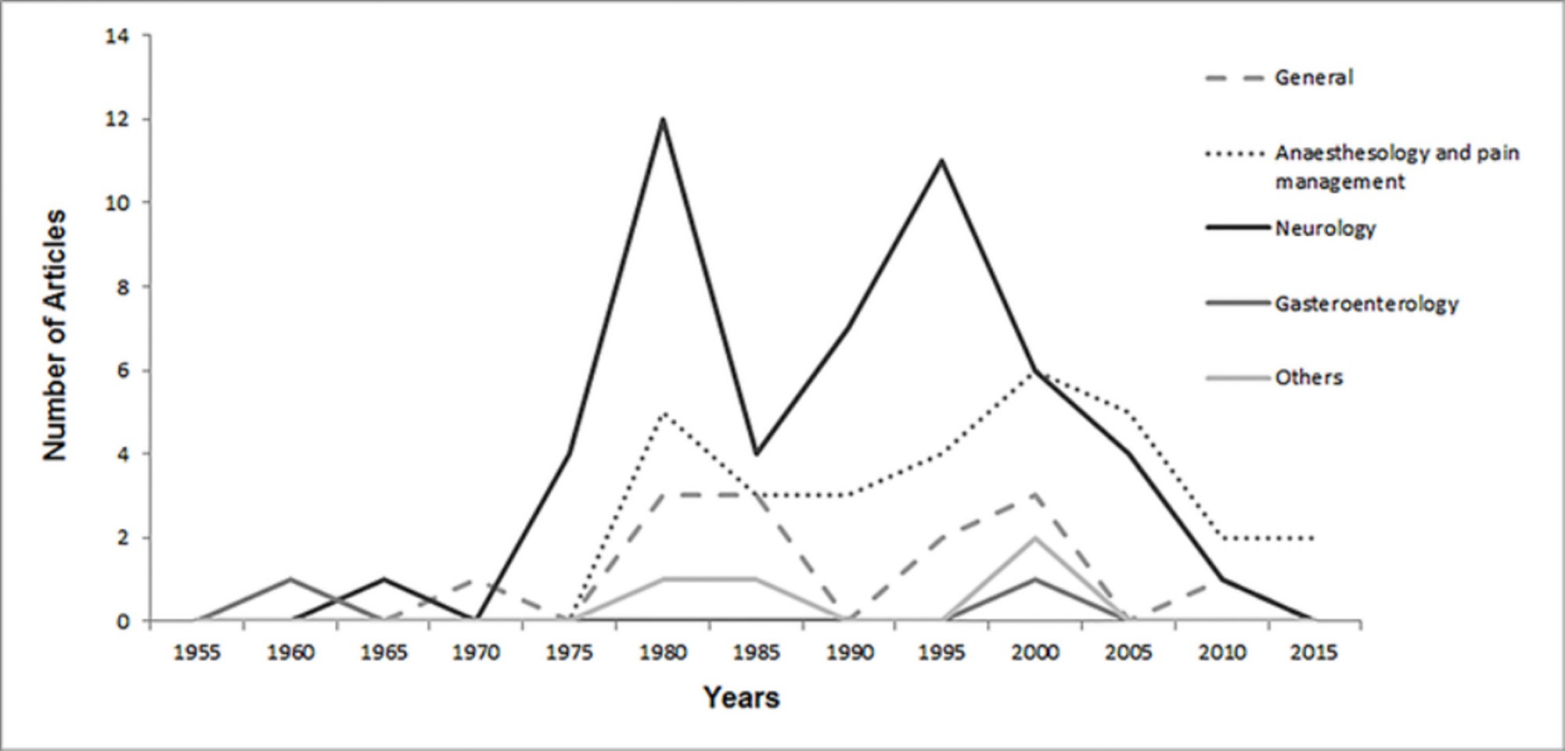
Number of articles published each year per major subject area.

Citation classics belonged to four major subject areas: neurosciences, anesthesiology and pain management, gastroenterology, and general medicine. Figure 3 shows the number of articles published each year by specific subject area. The distribution of citation count was same in all subject areas (p=0.260). No significant difference was found between subject areas and citation density (p=0.136) and year of publication (p=0.105).

Forty-six of the citation classics primarily discussed opioids’ mechanism of action, followed by adverse effects (n=24), clinical uses (n=13), pharmacokinetics (n=4), basic sciences (n=3), and opioid tolerance (n=2). The remaining seven discussed various other topics.

Classes of opioids 

**Figure 4:**
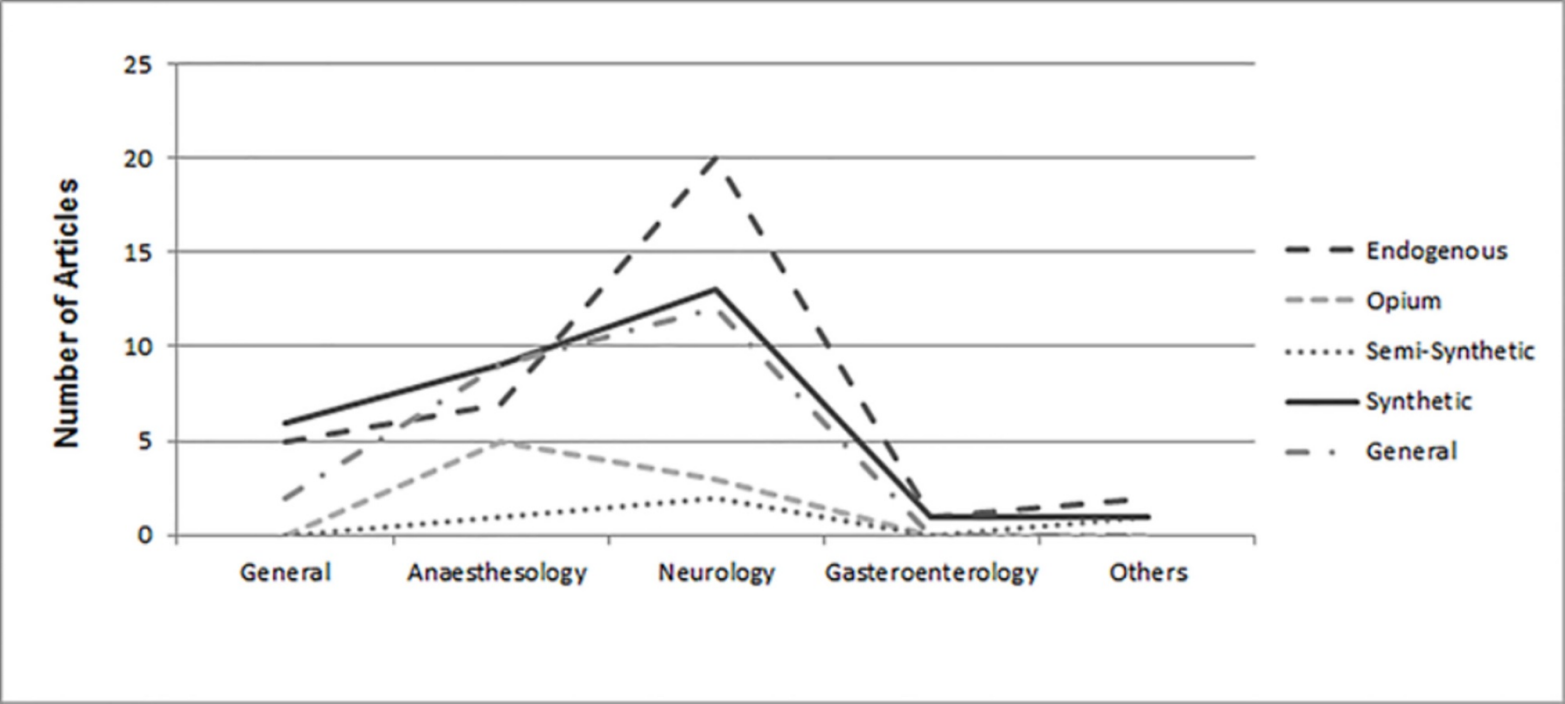
Number of articles on classification of opioids published in each subject area.

Citation classics were distributed among four major opioid classes and a miscellaneous topics category. Thirty-five articles discussed endogenous opioids, followed by synthetic opioids (n=30), opium (n=8), and semi-synthetic opioids (n=4). The remaining 23 citation classics discussed opioids in general. Significant differences were observed between classes of opioids and years of publication (p=0.037), citation count (p=0.000), and citation density (p=0.000). Figure 4 shows the number of articles on classification of opioids published in each subject area. There was no significant difference between articles published on different classes of opioids and subject areas (p=0.491).

## Discussion

The use of Opium dates back to the commencement of the Greek era [16]; opioids are opium derivatives [17]. The oldest opioid articles available on PubMed were published in 1945. The fact that none of these pioneer articles is a citation classic suggests that the quality of the work plays a greater role in citation number than does the number of years for which the article has been a part of the literature [12]. 

Trends of citation classics

Citation classics ranged over 53 years of publication, with zero to seven articles per year. Seventy-two of the citation classics were published from 1977 to 1997. The most-often cited article in the list was published in 1988. In contrast with previous studies [7], our graph of the total article citations over time (Figure 1) showed several peaks and troughs. After 1995, a gradual decrease in citations occurred, followed by a rapid decrease in 1997. Only 24 of all articles published after 1997 were citation classics. This finding supports the idea that some topics undergo intense study at certain times during which extensive research is performed, and after which the topic ceases to be of broad and current interest [18]. There was no significant difference between the citations of original research articles and review articles (p=0.310), contradicting the belief that review articles are more often cited [7]. Interestingly, only two articles had a citation density greater than one hundred, and the article ranked seventeenth had the highest citation density of 147.63 by Chou R et al. 

Origins, institutions and authorships

Sixty-three of the citation classics came from the United States (Figure 1). Campbell explains this major contribution from the USA by stating that reviewers and authors from the USA show bias towards local papers [19]. The United Kingdom produced the second-highest number of citation classics (n=13), followed by France (n=10). Thirteen of the citation classics were produced through the contributions of researchers in multiple countries.

In total, 160 institutions contributed to our citation classics. Forty institutions had more than two articles among the citation classics. Thirty-five of the citation classics had multi-institutional origins. Of these, 13 papers were also multinational, suggesting that international collaborations produce high-quality output that greatly benefits the scientific community [7].

A total of 500 authors contributed to the citation classics. Each of the top 12 authors contributed to at least three articles. Bloom F, although 12th in rank, had the highest H-index, followed by Watson SJ, Kosterlitz HW, and Akil H. Authors with a high H-index not only have a greater chance of having their work accepted, but are also more likely to get promotions and become reviewers [20].

Journals

As the results indicated, the citation classics were published in 40 journals. The top three journals were multidisciplinary and contained 36 citation classics. Sixteen were neurosciences-based journals with 23 citation classics. Seven were medicine journals with 13 citation classics. Similarly, pharmacology-based journals (n=7) had 12 citation classics, and anesthesiology journals had 11 citation classics. Among the multidisciplinary journals, Science had the highest CiteScore of 14.39 and the highest number of citation classics. Both top CiteScore journals had only one article each in the top hundred. Among the neuroscience-based journals, Annual Review of Neuroscience had the highest CiteScore of 16.43, followed by Trends in Neurosciences, with the CiteScore of 10.37. The top citation classic was published in Proceedings of the National Academy of Sciences of the United States of America, a multidisciplinary journal with a CiteScore of 8.56. This journal had ten articles in the list of citation classics (Table 2). Physiological Reviews, a medicine-based journal, had the highest CiteScore (29.60) overall, but only one citation classic was published in it.

According to the Bradford law, our top three multidisciplinary journals that had the highest citations are Zone 1 or core journals [21]. Similarly, the journals belonging to the categories of neurosciences and medicine are Zone 2 and Zone 3 journals, respectively. This trend indicates that although opioids are part of the field of pharmacology, citation classics on opioids were primarily published in multidisciplinary and neurology-based journals, which contradicts an established concept that highly cited articles are published in field-specific journals [13]. We used multiple analytical parameters to rank our journals, including CiteScore, SJR, and SNIP, to reduce bias [22]. CiteScore is a metric similar to a journal’s Impact Factor that gives us a comprehensive view of the journal’s effect on the scientific community.

Subject area and topical distribution

Neurosciences seemed to be the most studied subject area, as half of the citation classics covered topics in neurology, psychology, cognitive sciences, and psychiatry. This finding is similar to previous reports [23]. The most likely explanation is that the mechanism of action, clinical uses, tolerance, and addiction involve the neural system. Thirty-one of the citation classics addressed anaesthesiology and pain management, demonstrating the major role of opioids in this subject area. Even though opioids have major effects on the gastrointestinal tract [24], we observed that gastroenterology articles were underrepresented among the opioid citation classics. The work in various subject areas was uniformly distributed among years of publication.

As mentioned above, citation classics primarily discussed the opioid mechanism of action, covering the structure, saturation, availability, binding sites, and location of opioid receptors in various organs. Although deaths due to opioid overuse and addiction continue to skyrocket [25], 24 articles focused on the adverse effects of opioids. In these articles, drug dependence, abuse, and addiction were highlighted. Only one article among the citation classics examined prescription protocols.

Classes of opioids

Opioids are classified as endogenous, opium alkaloids, synthetic opioids, and semi-synthetic opioids [16]. Endogenous opioids, which include endorphins, enkephalins, and dynorphins, were most-studied. Twenty of the 35 citation classics regarding endogenous opioids fell under the subject area of neurology (Figure 4). The second most cited class was synthetic opioids. Thirteen of these 30 citation classics fell under the subject area of neurology. 

Some limitations must be considered. Possible citation bias, including in-house citations, negative citations, and incomplete citations is a major limitation. In addition, the use of one database to extract the list may have overlooked articles that were not recognized by Scopus, and excluded textbooks. Scopus has been reported to miss older citations, which results in omissions of research conducted and published before 1980 [26, 27]. Our list may have missed some citation classics; this can be explained as ‘obliteration by incorporation’ [12], which in simple terms states that the content of some classic articles has become such common knowledge that they no longer require citation.

## Conclusions

We conclude that the neurologic and analgesic aspects of opioids are well established in the top 100 citation classics. Endogenous and synthetic opioids were the most studied. Although addiction and misuse of opioids is a worldwide menace to health and economy, this concern was underrepresented in the citation classics. We would also like to draw attention to the pulmonary and gastrointestinal aspects of opioids, which are marginalized among the citation classics. Similarly, quality papers on opium alkaloids, semi-synthetic opioids, and substance abuse are needed.

## Appendices

**Table 3:** Top 100 Articles on opioids. CNS - Central Nervous System NMDA receptor - N-methyl-D-aspartate receptor COMT - Catechol-O-methyltransferase EAPC - European Association for Palliative Care

Rank	Title	Citation	Citation density	Most Cited (Year)
1	Drugs abused by humans preferentially increase synaptic dopamine concentrations in the mesolimbic system of freely moving rats. Proceedings of the National Academy of Sciences, 1988	3149	108.59	2014
2	Identification of two related pentapeptides from the brain with potent opiate agonist activity. Nature, 1975	2677	63.74	1977
3	The effects of morphine-and nalorphine-like drugs in the nondependent and morphine-dependent chronic spinal dog. Journal of Pharmacology and Experimental Therapeutics, 1976	2336	56.98	1982
4	A psychomotor stimulant theory of addiction. Psychological Review, 1987	2077	69.23	1999
5	Endogenous opioid peptides: multiple agonists and receptors. Nature, 1977	1928	48.2	1982
6	The formalin test: a quantitative study of the analgesic effects of morphine, meperidine, and brain stem stimulation in rats and cats. Pain, 1977	1843	46.08	2011
7	A randomized, controlled trial of methylprednisolone or naloxone in the treatment of acute spinal-cord injury: results of the Second National Acute Spinal Cord Injury Study. The New England Journal of Medicine, 1990	1820	67.41	2002
8	Drugs of abuse: anatomy, pharmacology and function of reward pathways. Trends in Pharmacological Sciences, 1992	1729	69.16	1998
9	Isolation and structure of the endogenous agonist of opioid receptor-like ORL1 receptor. Nature, 1995	1636	74.36	2000
10	Cellular and molecular mechanisms of drug dependence. Science, 1988	1606	55.38	2000
11	A neuropeptide that activates an opioidlike G protein-coupled receptor. Science, 1995	1592	72.36	2000
12	Intrathecal morphine in mice: a new technique. European Journal of Pharmacology, 1980	1433	38.73	2013
13	The self-medication hypothesis of addictive disorders: focus on heroin and cocaine dependence. In: The Cocaine Crisis: Springer; 1987	1413	44.16	2011-2012
14	Opiate receptor: demonstration in nervous tissue. Science, 1973	1367	31.07	1981
15	The formalin test in mice: dissociation between inflammatory and non-inflammatory pain. Pain, 1987	1355	45.17	2011;2014
16	Drug dependence, a chronic medical illness: implications for treatment, insurance, and outcomes evaluation. Jama, 2000	1291	75.94	2014
17	Clinical guidelines for the use of chronic opioid therapy in chronic noncancer pain. The Journal of Pain, 2009	1181	147.63	2011
18	Loss of morphine-induced analgesia, reward effect and withdrawal symptoms in mice lacking the mu-opioid-receptor gene. Nature, 1996	1139	54.24	2001
19	beta-Endorphin and adrenocorticotropin are selected concomitantly by the pituitary gland. Science, 1977	1101	27.53	1982
20	A potent and selective endogenous agonist for the mu-opiate receptor. Nature, 1997	1082	54.1	1999
21	Cloning of a delta opioid receptor by functional expression. Science, 1992	1028	41.12	1994
22	P-glycoprotein in the blood-brain barrier of mice influences the brain penetration and pharmacological activity of many drugs. Journal of Clinical Investigation, 1996	1020	48.57	2004
23	Opioids excite dopamine neurons by hyperpolarization of local interneurons. Journal of Neuroscience, 1992	1014	40.56	2011
24	Chronic parkinsonism secondary to intravenous injection of meperidine analogues. Psychiatry Research, 1979	1011	26.61	1986
25	Inhibition of morphine tolerance and dependence by the NMDA receptor antagonist MK-801. Science, 1991	1006	37.26	1996
26	Bispectral analysis measures sedation and memory effects of propofol, midazolam, isoflurane, and alfentanil in healthy volunteers. Anesthesiology, 1997	989	49.45	2006
27	Placebo and opioid analgesia--imaging a shared neuronal network. Science, 2002	969	64.6	2009
28	Unresponsiveness to cannabinoids and reduced addictive effects of opiates in CB1 receptor knockout mice. Science, 1999	960	53.33	2005
29	Anatomy of CNS opioid receptors. Trends in Neurosciences, 1988	954	32.9	1994
30	Opioid-receptor mRNA expression in the rat CNS: anatomical and functional implications. Trends in Neurosciences, 1995	936	42.55	1999;2000
31	Endogenous opioids: biology and function. Annual Review of Neuroscience, 1984	916	27.76	1988
32	Common precursor to corticotropins and endorphins. Proceedings of the National Academy of Sciences, 1977	913	20.83	1980
33	Heroin addicts have higher discount rates for delayed rewards than non-drug-using controls. Journal of Experimental Psychology: General, 1999	911	50.61	2016
34	Dynorphin is a specific endogenous ligand of the kappa opioid receptor. Science, 1982	906	25.89	1983
35	Opioid and nonopioid components independently contribute to the mechanism of action of tramadol, an 'atypical' opioid analgesic. Journal of Pharmacology and Experimental Therapeutics, 1992	898	35.92	2011
36	ORL1, a novel member of the opioid receptor family: cloning, functional expression and localization. FEBS letters, 1994	897	39	2000
37	Management of pain in elderly patients with cancer. Jama, 1998	896	47.16	2006
38	Role of unconditioned and conditioned drug effects in the self-administration of opiates and stimulants. Psychological Review, 1984	895	27.12	1989
39	The delta-opioid receptor: isolation of a cDNA by expression cloning and pharmacological characterization. Proceedings of the National Academy of Sciences, 1992	881	35.24	1994
40	Intrathecal and epidural administration of opioids. Anesthesiology, 1984	837	25.36	1989
41	Lack of analgesic effect of opioids on neuropathic and idiopathic forms of pain. Pain, 1988	826	28.48	2003
42	COMT val158met genotype affects µ-opioid neurotransmitter responses to a pain stressor. Science, 2003	820	58.57	2009
43	Endogenous pain control mechanisms: review and hypothesis. Annals of Neurology, 1978	793	20.33	1984
44	Molecular cloning and functional expression of a mu-opioid receptor from rat brain. Molecular Pharmacology, 1993	788	32.83	1996
45	Morphine, gabapentin, or their combination for neuropathic pain. The New England Journal of Medicine, 2005	787	65.56	2011
46	Single-nucleotide polymorphism in the human mu opioid receptor gene alters β-endorphin binding and activity: possible implications for opiate addiction. Proceedings of the National Academy of Sciences, 1998	783	41.21	2013
47	Stereospecific binding of the potent narcotic analgesic [3H] etorphine to rat-brain homogenate. Proceedings of the National Academy of Sciences, 1973	770	17.5	1981
48	Bis-penicillamine enkephalins possess highly improved specificity toward delta opioid receptors. Proceedings of the National Academy of Sciences, 1983	767	22.56	1991
49	Cannabinoid and heroin activation of mesolimbic dopamine transmission by a common µ1 opioid receptor mechanism. Science, 1997	757	37.85	2007
50	Autoradiographic differentiation of mu, delta, and kappa opioid receptors in the rat forebrain and midbrain. Journal of Neuroscience, 1987	748	24.93	1994
51	U-50,488: a selective and structurally novel non-Mu (kappa) opioid agonist. Journal of Pharmacology and Experimental Therapeutics, 1983	732	21.53	1991
52	Opposite effects of mu and kappa opiate agonists on dopamine release in the nucleus accumbens and in the dorsal caudate of freely moving rats. Journal of Pharmacology and Experimental Therapeutics, 1988	723	24.93	1997
53	Dynorphin-(1-13), an extraordinarily potent opioid peptide. Proceedings of the National Academy of Sciences, 1979	722	19	1982
54	Dissociable deficits in the decision-making cognition of chronic amphetamine abusers, opiate abusers, patients with focal damage to prefrontal cortex, and tryptophan-depleted normal volunteers: evidence for monoaminergic mechanisms. Neuropsychopharmacology, 1999	721	40.06	2005
55	Opioids in chronic non-cancer pain: systematic review of efficacy and safety. Pain, 2004	719	55.31	2010
56	Opposing tonically active endogenous opioid systems modulate the mesolimbic dopaminergic pathway. Proceedings of the National Academy of Sciences, 1992	713	28.52	2012
57	Opiate analgesics inhibit substance P release from rat trigeminal nucleus. Nature, 1977	702	17.55	1984
58	Influence of age and gender on the pharmacokinetics and pharmacodynamics of remifentanil. Model Development. The Journal of the American Society of Anesthesiologists, 1997	699	34.95	2013;2014
59	Progressive ratio schedules in drug self-administration studies in rats: a method to evaluate reinforcing efficacy. Journal of Neuroscience Methods, 1996	698	33.24	2015
60	The Severity of Dependence Scale (SDS): psychometric properties of the SDS in English and Australian samples of heroin, cocaine and amphetamine users. Addiction, 1995	696	31.64	2016
61	Mechanisms of hyperalgesian and morphine tolerance: a current view of their possible interactions. Pain, 1995	694	31.55	2005
62	The effects of psychosocial services in substance abuse treatment. Addictions Nursing Network, 1993	691	28.79	2004
63	Morphine and alternative opioids in cancer pain: the EAPC recommendations. British Journal of Cancer, 2001	688	43	2011
64	A medical treatment for diacetylmorphine (heroin) addiction: a clinical trial with methadone hydrochloride. Jama, 1965	679	13.06	1971
65	Tolerance of locus coeruleus neurones to morphine and suppression of withdrawal response by clonidine. Nature, 1978	675	17.31	1996
66	Immunohistochemical localization of enkephalin in rat brain and spinal cord. Journal of Comparative Neurology, 1978	673	17.26	1983
67	Autoradiographic localization of opiate receptors in rat brain. Spinal cord and lower medulla. Brain Research, 1977	665	16.63	1982
68	Opioid therapy for chronic pain. The New England Journal of Medicine, 2003	659	47.07	2010
69	Isolation of an endogenous compound from the brain with pharmacological properties similar to morphine. Brain Research, 1975	657	15.64	1977
70	Opioid-induced hyperalgesia: a qualitative systematic review. The Journal of the American Society of Anesthesiologists, 2006	648	58.91	2012
71	The action of morphine and related substances on contraction and on acetylcholine output of coaxially stimulated guinea‐pig ileum. British Journal of Pharmacology, 1957	632	10.53	1980
72	Acute opioid tolerance intraoperative remifentanil increases postoperative pain and morphine requirement. The Journal of the American Society of Anesthesiologists, 2000	630	37.06	2015
73	Intravenous cocaine, morphine, and amphetamine preferentially increase extracellular dopamine in the" shell" as compared with the" core" of the rat nucleus accumbens. Proceedings of the National Academy of Sciences, 1995	624	28.36	1999
74	A new method for receptor autoradiography: [3 H] opioid receptors in rat brain. Brain Research, 1979	620	16.32	1985
75	Organization of endogenous opiate and nonopiate pain control systems. Science, 1982	609	17.4	1986
76	Impulsivity as a vulnerability marker for substance-use disorders: review of findings from high-risk research, problem gamblers and genetic association studies. Neuroscience & Biobehavioral Reviews, 2008	608	67.56	2014
77	Neurobiology of relapse to heroin and cocaine seeking: a review. Pharmacological Reviews, 2002	608	40.53	2005
78	Opioid and nonopioid mechanisms of stress analgesia. Science, 1980	606	16.38	1983
79	Cellular and synaptic adaptations mediating opioid dependence. Physiological Reviews, 2001	604	37.75	2006
80	Crystal structure of the [micro]-opioid receptor bound to a morphinan antagonist. Nature, 2012	594	118.8	2013
81	The enkephalinase inhibitor thiorphan shows antinociceptive activity in mice. Nature, 1980	594	16.05	1989
82	Endorphins: profound behavioral effects in rats suggest new etiological factors in mental illness. Science, 1976	587	14.32	1979
83	Halothane–morphine compared with high-dose sufentanil for anesthesia and postoperative analgesia in neonatal cardiac surgery. The New England Journal of Medicine, 1992	584	26.55	2001
84	Control CfD, Prevention: Vital signs: overdoses of prescription opioid pain relievers - United States, 1999 - 2008. MMWR Morbidity and Mortality Weekly Report, 2011	582	97	2016
85	Immunohistochemical studies using antibodies to leucine-enkephalin: initial observations on the nervous system of the rat. Neuroscience, 1976	577	14.07	1980
86	The neostriatal mosaic: compartmentalization of corticostriatal input and striatonigral output systems. Nature, 1984	571	17.3	1989
87	Cloning and sequence analysis of cDNA for porcine β-neo-endorphin/dynorphin precursor. Nature, 1982	571	16.31	1986
88	Classification of opioid receptors. 1983.	570	16.76	1986
89	Pain relief by intrathecally applied morphine in man. Survey of Anesthesiology, 1979	570	15	1982
90	Addictive drugs and brain stimulation reward. Annual Review of Neuroscience, 1996	569	27.1	2005
91	Self-administration of psychoactive substances by the monkey. Psychopharmacology, 1969	566	11.79	1976
92	Efficacy of oxycodone in neuronathic pain: a randomized trial in postherpetic neuralgia. Neurology, 1998	565	29.74	2003
93	Clinical pharmacology of tramadol. Clinical Pharmacokinetics, 2004	563	43.31	2016
94	Multiple opiate receptors. Enkephalins and morphine bind to receptors of different specificity. Journal of Biological Chemistry, 1979	563	14.82	1982
95	International Union of Pharmacology. XII. Classification of opioid receptors. Pharmacological Reviews, 1996	561	26.71	1999
96	Cloning and functional comparison of kappa and delta opioid receptors from mouse brain. Proceedings of the National Academy of Sciences, 1993	561	23.38	1996
97	Mu, delta, and kappa opioid receptor mRNA expression in the rat CNS: an in situ hybridization study. Journal of Comparative Neurology, 1994	557	24.22	1999
98	Intrathecal morphine inhibits substance P release from mammalian spinal cord in vivo. Nature, 1980	551	14.89	1988
99	Opioid complications and side effects. Pain Physician Journal, 2008	545	60.56	2016
100	Neurobiological similarities in depression and drug dependence: a self-medication hypothesis. Neuropsychopharmacology, 1998	544	28.63	2002
